# Brain changes with Chinese reading development in typical and atypical readers

**DOI:** 10.3389/fpsyg.2023.1292985

**Published:** 2023-11-21

**Authors:** Fan Cao

**Affiliations:** ^1^Department of Psychology, The University of Hong Kong, Pok Fu Lam, Hong Kong SAR, China; ^2^State Key Lab of Brain and Cognitive Sciences, The University of Hong Kong, Pok Fu Lam, Hong Kong SAR, China

**Keywords:** reading development, reading disability (dyslexia), Chinese, fMRI, brain development

## Abstract

Reading is a high-order cognitive process that is unique in human beings. There is a prolonged developmental course and a wide range of proficiency levels associated with reading. In this review, I focus on brain changes underlying Chinese reading development in both typical readers and readers with reading disability. Reading development in typical readers is characterized by a shift from dorsal phonological reading to ventral orthographic reading in the brain and increased interactive specialization in the reading network. Even though some individuals with reading disability may be able to catch up with typical readers on phonological reading by adulthood, they cannot reach fluent orthographic reading. In the brain, the reduction of brain activation in the left inferior frontal gyrus associated with reading disability disappears by adulthood, suggesting that this is a developmental delay, while there is a greater reduction of brain activation in the left inferior temporal gyrus in adults than children with reading disability. It suggests a greater deficit in the dorsal phonological reading pathway in children and a greater deficit in the ventral orthographic reading pathway in adults with reading disability. This review provides insights about the developmental trajectories in typical and atypical reading.

## Introduction

Learning to read is one of the most important missions in child development. Significant structural and functional brain changes are accompanied with learning to read, and studying reading development in the developing brain offers a great opportunity to understand how learning experience interacts with brain maturation. Moreover, in comparison to typical readers, individuals with reading disability experience an atypical developmental trajectory of reading both behaviorally and neurologically. It is of great interest to understand how learning and development take place in the atypical brain. However, very few studies and reviews have concerned brain changes during reading development in readers with reading disability after formal schooling, and there is even less such research in Chinese reading disability. Chinese, a very contrastive language comparing to alphabetic languages, offers a chance to understand the generality and specificity in reading development and disorders across different languages. Chinese is a morpho-syllabic language, in which each character represents a morpheme and a syllable rather than a word. Chinese characters have more complex visual-spatial layout than alphabetic languages, in which strokes are packed in a square with top-down, left-right and inside-outside spatial arrangement of the strokes. Moreover, strokes do not map to phonemes in the syllable and the whole character has to be mapped to the whole syllable. Taken together, morphological skills and holistic visual orthographic configuration skills might be more essential than phonological skills in Chinese reading development and disability due to the specific features of Chinese.

## Reading development in typical readers

Reading is essentially mapping visual symbols to the spoken language system to acquire meaning. According to Ehri (1995, 2014), there are three stages in reading development, namely, logographic reading, phonological reading and orthographic reading (Ehri, 1995, 2014). Logographic reading is when young children memorize the meaning of a word based on the shape of letters and word. This stage cannot be considered as real reading yet, because it is based on arbitrary connections between word shape and meaning. Phonological reading is when children apply grapheme-phoneme-correspondence to convert scripts to sounds and then access meaning. This stage is when children learn to read. The orthographic reading stage is when readers can recognize the sight words from memory without effortful grapheme-phoneme-correspondence. This is the stage when children read to learn, because attention can be allocated to comprehension rather than word recognition. Corroborating evidence from behavioral studies in alphabetic languages has supported this reading developmental model.

No developmental models have been proposed to explain Chinese reading development; however, studies have shown developmental changes in Chinese reading. For example, two proof-reading studies have found that children show a greater reliance on phonology while adults show a greater reliance on orthography during Chinese reading (Peng et al., 1985; Song et al., 1995), suggesting that children tend to convert orthography to phonology then access meaning while adults tend to rely on the direct mapping from orthography to semantics. In these studies, participants were asked to identify spelling mistakes when reading a paragraph. The spelling mistakes were either homophones of the target character or orthographically similar to the target character. Children identified less homophone mistakes than adults while adults identified less orthographic mistakes than children, suggesting that children rely more on phonological information whereas adults rely more on orthographic information. Taken together, during the early stage of Chinese reading acquisition, phonology plays an important role in bridging the print with meaning, however, with increased reading proficiency, the print can be directly connected to meaning with phonology simultaneously activated (Perfetti et al., 2005). It is important to note that the phonological reading and orthographic reading are by no means exclusive, rather both reading processes exist in most readers.

Furthermore, there is an even greater reliance on the direct mapping from orthography to semantics in proficient reading in Chinese than in English, due to the morpho-syllabic nature of the Chinese writing system. Each Chinese character represents a syllable and a morpheme with the existence of substantial homophones. Therefore, phonology is not reliable in mapping to meaning and the direct mapping from orthography to semantics is more efficient. Consistent with these features of Chinese, studies have also found that morphological awareness plays a more important role in reading development than phonological awareness, especially in later stage of Chinese reading development (McBride and Wang, 2015; Song et al., 2019). Another study found that children with greater morphological awareness at the age of 4.5 years had a faster growth rate in reading between 4.5 and 6 years of age (Lin et al., 2019). Visual attention span was found to play an essential role in reading fluency in Chinese children adolescents and adults (Huang et al., 2019), suggesting that the complex visual configuration of Chinese characters may have a great demand on visual attention span.

## Brain changes underlying typical reading development

Consistent with the reading developmental shift from phonological reading to orthographic reading, there is a dorsal to ventral shift in the reading brain (Pugh et al., 2000). One important region in the dorsal reading pathway is the left temporo-parietal area which is involved in phonological representation and phonological assembly, while one important region in the ventral reading pathway is the left inferior temporal gyrus which is involved in rapid orthographic recognition and sight word reading (Pugh et al., 2000). Neuroimaging research on both English and Chinese has shown that there is a developmental decrease in the activation of the left superior temporal gyrus (Bitan et al., 2007; Cao et al., 2010) and a developmental increase in the left inferior temporal gyrus during visual word tasks (Booth and Burman, 2005; Cao et al., 2010; Ben-Shachar et al., 2011), consistent with the dorsal phonological reading to ventral orthographic reading shift model (Martin et al., 2015). In addition, the right middle occipital gyrus has also been found to be more involved in Chinese adults than Chinese children (Cao et al., 2010, 2015), suggesting the importance of holistic visual configuration in Chinese word reading. Two recent studies using graph theory analysis found increased connectivity strength and nodal degree in vision-related and semantic-related regions but decreased connectivity in phonology-related regions in Chinese adults than in Chinese children (Liu et al., 2018; Zhou et al., 2021), consistent with the phonological reading to orthographic reading shift.

The interactive specialization hypothesis (Johnson, 2005) has been proposed to explain brain mechanisms underlying expertise acquisition and brain development, which argues that skill acquisition and brain development are characterized by increased functional specialization and interactivity between brain regions. Specifically, when a certain brain region is functionally specialized, it becomes only involved in relevant neural computations and uninvolved in irrelevant computations. Brain changes during reading development have been found to be consistent with the interactive specialization hypothesis. For example, it was found that children showed greater overlap than adults in brain activation between visual word tasks and auditory word tasks, whereas adults showed greater activation in the left fusiform gyrus for visual word tasks and greater activation in the left superior temporal gyrus for auditory word tasks, suggesting greater modality specialization in adults than in children (Booth and Burman, 2005). Furthermore, Chinese research also supports the interactive-specialization hypothesis. For example, one study has found task differentiation in the brain activation in the left inferior parietal lobule which was more activated in a phonological task than in a semantic task in Chinese adults but not in children, suggesting that there is greater specialization for phonological processing in this region in Chinese adults than in children (Cao et al., 2009). Another study found that only adults but not children showed task differences in inter-regional connectivity using graph theory (Liu et al., 2018), suggesting task specialization in adults but not children. A study on 7–64 years old Chinese speakers found age-related decrease in brain activation in the Chinese reading network, suggesting a more focused and specialized reading network (Siok et al., 2020), supporting the interactive specialization theory.

In addition, studies have also suggested increased interactivity between brain regions with reading development. For example, a study found greater connectivity in the prefrontal-superior temporal network in adults than children in audiovisual integration during Chinese reading (Li et al., 2023). Another study found age-related increase in the functional connection of the Chinese reading network during visual semantic processing (Jia et al., 2023). To summarize, with reading development, brain regions in the reading network become more specialized as well as more interconnected.

## Reading disability

Unfortunately, about 7% of the population experience reading disability across different languages in the world according to a recent meta-analysis study (Yang et al., 2022), which is a significant reading impairment despite appropriate cognitive ability, learning motivation and opportunity (Shaywitz et al., 1995). Reading disability (RD) is defined as a developmental neurological disorder with a genetic origin, however, the complexity of gene-brain-behavior relationships hinders our understanding of the exact etiologies of RD. After decades of research on RD, phonological deficit hypothesis appears to be one of the most compelling theories, which argues that deficiency in phonological representation, retrieval and manipulation hinders the letter-sound mapping in reading acquisition (Snowling and Melby-Lervag, 2016). However, evidence has not been strong enough to support a causal relationship between phonological deficit and RD (Siok and Tan, 2022), since there are cases when poor phonological skills are associated with normal reading (Snowling et al., 2000) and intact phonological skills are associated with poor reading (Manis et al., 1995). It appears that multifaceted deficits are associated with reading disability and a single deficit cannot explain the heterogeneity of RD (Perry et al., 2019). Moreover, in addition to phonological awareness, morphological awareness, orthographic skills and rapid automatized naming have also been found to be cognitive cores in Chinese reading development and disorders (McBride and Wang, 2015).

## Brain functional differences associated with RD

Neuroimaging research has revealed altered brain functions associated with RD. For example, reduced brain activation in the left occipito-temporal and left temporo-parietal areas has been reported (Richlan, 2012), suggesting deficiency in the orthographic or phonological processing, respectively. Even though many neuroimaging studies have shown evidence of phonological deficits in the brain in individuals with RD in both English (Shaywitz et al., 1998) and Chinese (Cao et al., 2017), a recent Chinese study, using a dynamic causal modeling approach, found that during reading, the phonological deficits were actually due to reduced input from the orthographic region (Yan et al., 2021b). It suggests that deficient orthographic processing and/or weak connection from orthography to phonology are indispensable in Chinese reading failure. Another study also found increased neural noise during visual word spelling judgment but not during auditory word rhyming judgment using a multivariate pattern analysis method (Wu et al., 2022). Taken together, it suggests that orthographic deficits may be as salient as or even more salient than phonological deficits in Chinese RD.

As for the left inferior frontal gyrus (IFG), however, there is less consensus about its abnormality in the literature of RD (Danelli et al., 2017). Some studies found reduced activation in this region in individuals with RD than in typical controls, while other studies found increased activation in this region instead. The inconsistency might be due to various task difficulties and participants’ ages. For easier tasks and adult participants, there tends to be increased activation in the left IFG in individuals with RD compared to typical controls, while for difficult tasks and child participants, there tends to be reduced activation. For easier tasks and more experienced readers with RD, increased activation in the left IFG has usually been interpreted as a compensation mechanism using articulatory rehearsal (Shaywitz et al., 1998). Compensations in individuals with RD have also been repeatedly found in the right hemisphere (Martin et al., 2016).

In addition to the task difficulty and age issues, cross-linguistic differences may also explain the inconsistent findings about the left IFG across studies. A recent meta-analysis study on hundreds of previous studies has focused on language differences in brain structural and functional alterations associated with RD (Yan et al., 2021a). It revealed greater structural and functional reduction in the left IFG in Chinese RD than in alphabetic languages, whereas there are greater alterations in the left fusiform gyrus in alphabetic languages than in Chinese. The language-specific alterations might represent consequent difficulties in learning to read a specific language with RD, while language-universal alterations might represent causes of RD. Chinese, as a non-alphabetic language, does not have grapheme-phoneme-correspondence rules and relies on whole-character-to-whole-syllable mapping during reading, for which the left dorsal IFG is responsible (Tan et al., 2005). This region is more involved in Chinese reading than in alphabetic reading in typical adult readers (Bolger et al., 2005), which explains why there are greater deficits in this region in Chinese dyslexic readers than in alphabetic counterparts. A recent study suggest that reduced brain activation in the left IFG might be due to word decoding deficits in Chinese children with RD, because only the subtype of poor decoders showed reduced activation in this region, while the subtype of poor comprehension was associated with reduced activation in the left inferior temporal gyrus (ITG) (Feng et al., 2023).

## Reading development in atypical readers

The differences between children and adults with RD have been overlooked. Would they show a shift from phonological reading to orthographic reading? Would they show an increased interactive specialization with age? According to the limited studies, phonological awareness deficits seem to be a persistent problem in individuals with RD (Bruck, 1992; Del Tufo and Earle, 2020). Even though word reading accuracy can be improved, word reading fluency is still lower in adults with RD than typical controls, suggesting that reading speed is still a challenge in adults with RD (Carioti et al., 2021). A recent meta-analysis summarized symptoms in adults with RD and found that they are poor on all reading and writing tests, with speed measures even more pronounced, and phonological awareness is a mild problem in shallow orthographies (Reis et al., 2020).

Even though, in Chinese, no published studies have compared children and adults with RD either behaviorally or neurologically, one recent study has compared Chinese dyselxic children and control children on behavioral tests from grade 2 to grade 6 (Siok and Tan, 2022). It was found that the gap between typical controls and RD children increased with age on a character reading test, decreased on RAN and working memory measures, and remained consistent on phonological awareness and orthographic awareness measures. These are very important findings for people to understand the prognosis of RD in Chinese. Moreover, in a recent study, it was found that there was a greater discrepancy between RD adults and control adults than that between RD children and control children on sentence reading fluency (Yan et al., under review). This is because typical readers have a greater development on sentence reading fluency than readers with RD, suggesting that “the rich get richer.” Sentence reading fluency is a comprehensive measure of reading proficiency which taps into word decoding, reading comprehension and speed. The developmental trajectory of such a high-order reading skill is derailed by primary deficits in word decoding early in childhood. This study also found persistent deficits in phonological awareness and word reading fluency in Chinese children and adults with RD, however, word reading accuracy was improved in Chinese adults with RD and they showed no differences from control adults. Taken together, individuals with RD catch up on word decoding accuracy by adulthood, but they show persistent deficits in phonological awareness, and word decoding fluency and they show an even greater deficit in sentence reading fluency in adults than in children. Therefore, it seems that Chinese RD readers catch up on phonological reading (i.e., word decoding accuracy), but they cannot reach fluent orthographic reading (i.e., sentence reading fluency), under the framework of the reading developmental stages by Ehri, 1995. In summary, reading development in RD is characterized by an unsuccessful shift from phonological reading to orthographic reading.

## Brain changes underlying reading development in RD

There have been very few studies that have actually concerned brain developmental changes in individuals with RD. A meta-analysis study has collected studies on children and adults with RD and made a comparison (Richlan et al., 2011). It was found that the reduction of brain activation in the bilateral inferior parietal lobule was more evident in children than in adults with RD, while the reduction in the left temporo-occipital area was more evident in adults than in children with RD. Another study examined age-correlations in 5–20 years old typical and atypical readers, and it was found that typical readers showed a positive age correlation in the left ITG, while individuals with RD showed a positive age correlation in the left IFG (Shaywitz et al., 2007). Findings of these two studies are consistent with the developmental shift model by Pugh et al. (2000) that typical readers shift from dorsal phonological reading to ventral orthographic reading, so that the dorsal pathway is more impaired in children with RD, while the ventral pathway is more impaired in adults with RD. This is because the shift is not successful in individuals with RD.

No published studies have compared brain alterations in Chinese children and adults with RD. However, a recent study (Yan et al., under review) has examined this topic and found that Chinese adults with RD showed reduced brain activation than control adults in the left ITG while Chinese children with RD showed reduced brain activation than control children in the left IFG. Greater alterations in the left ITG in adults with RD and in the left IFG in children with RD have been replicated in three different tasks, namely, a visual rhyming task, a visual spelling task and an auditory rhyming task across different samples of participants. Further analyses revealed greater developmental increase in the left IFG in RD readers than in typical readers, and greater developmental increase in the left ITG in typical readers than RD readers. These findings from Chinese are consistent with those from the meta-analysis study and the age-correlation study in alphabetic languages.

According to this study (Yan et al., under review), it appears that RD readers can catch up on the left IFG but show increased impairment in the left ITG over development, suggesting a developmental delay in the left IFG but a developmental deviance in the left ITG. Consistent with this finding, another study also suggests different natures of alteration in the left IFG and left ITG in RD readers (Cao et al., 2021). It was found that children with RD showed no difference from a group of reading-matched younger control children in the brain activation in the left IFG, both of whom were lower than the age-matched control children in a visual rhyming judgment task, suggesting that this brain region is sensitive to reading level and/or task performance, rather than RD. When the reading level was matched, brain activation was matched, even though one group was RD and the other was not. However, in the left ITG, it was found that the reading control group was similar to the age control group, both of whom were higher than the RD group in brain activation, suggesting that this region is not sensitive to reading level but sensitive to RD *per se*. For typical children, even those younger ones with a lower reading skill, brain activation at this region was higher than RD children. Taken together, RD readers show a significant increase in phonological reading at the left IFG over development; however, they do not show adequate increase in orthographic reading in the left ITG.

Moreover, the catch-up at the left IFG seems to be driven by over-connectivity with other brain regions especially in the right hemisphere in adults with RD (Yan et al., under review). Previous studies have suggested that strong connection with other parts of the brain helps to normalize brain function of the left IFG (Richards et al., 2018). Additionally, it was also found that there is a lack of developmental increase in task specialization in RD readers (Yan et al., under review). During the auditory rhyming task, typical readers showed a development increase in bilateral STG but RD readers did not. For the auditory task, the bilateral STG play an essential role, and typical adult readers showed greater activation than typical child readers, suggesting increased task specialization. However, RD adults and children showed similar activation in this region. It suggests that reading development in RD readers is not characterized by increased task specialization, according to the interactive specialization hypothesis (Johnson, 2005), which might implicate possible different learning mechanisms in the atypical brain.

Findings of developmental changes in individuals with RD have important implications in the diagnosis and treatment of RD. For example, diagnosis and treatment may be focused on word reading accuracy in children but reading fluency in adults since we found children and adults with RD showed the most significant deficits in word reading accuracy and reading fluency, respectively. During reading treatment, brain stimulation at age-specific regions, such as the left IFG for children and left ITG for adults may be helpful, because we found reduced brain activation in the left IFG in children with RD and in the left ITG in adults with RD. Our findings provide important insights in developing age-specific and more individualized diagnosis and treatment programs for individuals with RD.

## Concluding remarks

Typical reading development is characterized by increased involvement of the ventral orthographic reading pathway and decreased involvement of the dorsal phonological reading pathway. It is also characterized by increased interactive specialization in the reading network, so that each region is only involved in certain computations and there is an increased connectivity between regions. Reading development in individuals with RD is characterized by developmental delay in word decoding accuracy, but persistent deficits in phonological awareness and word decoding fluency, and increased deficits in sentence reading fluency over development. In the brain, reading development in RD readers is characterized by developmental delay in phonological reading at the left IFG and a failure in shifting to the ventral orthographic reading in the left ITG. Atypical reading development is also characterized by a lack of developmental increase in task specialization, suggesting possible alternative learning mechanisms in the atypical brain.

## Author contributions

FC: Conceptualization, Funding acquisition, Writing – original draft, Writing – review and editing.
